# Impact of TBT-S intervention on fathers of young adults with anorexia nervosa in Greece

**DOI:** 10.1192/j.eurpsy.2026.12026

**Published:** 2026-07-31

**Authors:** M. Tsiaka, E. Dieti

**Affiliations:** Hellenic Center For Eating Disorders, Athens, Greece

## Abstract

**Introduction:**

Anorexia nervosa (AN) is a severe illness with significant medical risks and negative repercussions in family life. Parents of patients with AN report high levels of depression, anxiety, and expressed emotion, which refers to parents’ emotional responses toward patients, such as criticism and emotional overinvolvement. Moreover, parents engage in accommodating behaviors or even participate in eating rituals, which may initially provide reassurance to patients but eventually contribute to the maintenance of AN symptoms. Indeed, parental involvement is strongly recommended in the treatment of AN. Family-based interventions and parental skills trainings aim to reduce parental distress and provide parents with skills to support the patient. In the same line, Temperament-Based Treatment with Supports (TBT-S) is a multifamily, intensive intervention for patients with AN and their supports, which incorporates neurobiological and family dimensions. It involves psychoeducation on neurobiological underpinnings and temperamental traits in AN, meal implementation to practice skills to manage dysfunctional family interactions and redirecting expression of temperamental traits in a constructive manner to manage AN symptomatology. Previous findings indicate that parents reported improvements in family functioning, however differences between mothers and fathers regarding the impact of TBT-S intervention have not been investigated. Thus, fathers’ perspective remains largely unexplored.

**Objectives:**

The present study aimed to investigate the impact of the 4-day TBT-S intervention on 22 fathers of young adults with AN.

**Methods:**

Fathers completed questionnaires measuring psychological distress (Depression and Anxiety Stress Scale-21), expressed emotion (Family Questionnaire), and accommodating and enabling behaviors (Accommodation and Enabling Scale for Eating Disorders) immediately before and after the implementation of TBT-S intervention.

**Results:**

Fathers displayed significant increases in depression (mean ± SD: 10.45 ± 6.23 vs. 6.27 ± 4.59, *p* = .006) and anxiety (mean ± SD: 5.55 ± 5.23 vs. 3.45 ± 3.91, *p* = .038) compared to mothers who showed significant reductions in depression (mean ± SD: 10.00 ± 8.88 vs. 14.83 ± 9.14, *p* = .001), stress (mean ± SD: 14.28 ± 11.42 vs. 18.21 ± 9.01, *p* = .022), criticism (mean ± SD: 20.83 ± 5.46 vs. 23.79 ± 5.29, *p* < .001), and several accommodating and enabling behaviors. Further, a positive association emerged between changes in fathers’ depression and changes in mothers’ criticism (*r* = .45, *p* = .047) and accommodating and enabling behaviors specifically regarding rituals around meal context (*rho* = .50, *p* = .025).

**Conclusions:**

These findings indicate the diverse involvement of mothers and fathers in the treatment of AN and pinpoint the importance of inquiring more into the paternal role in the maintenance and treatment of AN.

**Disclosure of Interest:**

None Declared

